# Traces of Intentionality: Balance, Complexity, and Organization in Artworks by Humans and Apes

**DOI:** 10.1111/tops.70022

**Published:** 2025-09-01

**Authors:** Larissa M. Straffon, Juan O. Perea‐García, Tijmen den Blaauwen, Mariska E. Kret

**Affiliations:** ^1^ Cognitive Psychology Unit Leiden University; ^2^ Department of Psychosocial Science University of Bergen; ^3^ Center for Language Evolution Studies Nicolaus Copernicus University; ^4^ Department of Modern Philology University of Las Palmas de Gran Canaria; ^5^ Leiden Institute for Brain and Cognition (LIBC) Leiden University

**Keywords:** Abstract art, Intentionality, Chimpanzee art, Art perception, Art preference, Perceptual features

## Abstract

Are people able to tell apart a random configuration of lines and dots from a work of art? Previous studies have shown that untrained viewers can distinguish between abstract art made by professional artists, children, or apes. Pieces made by artists were perceived as more intentionally made and organized than the rest. However, these studies used paintings by prominent abstract artists (e.g., Mark Rothko) as stimuli, which in any case showed that people were able to recognize high‐quality paintings made by trained artists, not “any” human. In this study, we presented participants with artworks by untrained human artists versus artworks made by captive chimpanzees in a visual discrimination task. In Study 1, participants viewed sets of human‐ and non‐human‐made paintings and were asked to identify the artist as human or ape. In Study 2, they rated the paintings on several criteria: intentionality, organization, balance, and complexity. We found that participants: (1) successfully distinguished between human‐made and non‐human‐made paintings; (2) reported perceiving more balance, organization, and intentionality in human‐made paintings; (3) preferred stimuli, which ranked higher in intentionality. We also identified balance, complexity, and organization as key features that influence preference for abstract artworks. Overall, our results show that even non‐figurative paintings made by adults untrained in the visual arts are perceived as intentionally made, suggesting people spontaneously produce and perceive cues of intentionality, generating an implicit human signature in visual art.

## Abstract 2

This study investigates whether people can distinguish between abstract artworks made by untrained humans and those made by chimpanzees. Across two experiments, participants reliably identified human‐made works and rated them as more intentional, balanced, and organized. The findings suggest that humans, even without formal training, produce visual traces of intentionality that are perceptually recognized by others.

## 1 Introduction

Representational art usually depicts images of objects, landscapes, or people, which are easily identified by viewers, even when they are not particularly realistic, like in impressionist paintings. Abstract artworks such as the infamous splatter paintings by Jackson Pollock, in contrast, can be more challenging to the general public, who may perceive them as just random compositions of form and color (Alvarez, Winner, Hawley‐Dolan, & Snapper, 2015). Yet, a series of past studies have demonstrated that people, even at a young age, are somehow able to distinguish between professional abstract paintings and very similar pieces made by children and great apes (Hawley‐Dolan & Winner, 2011; Nissel, Hawley‐Dolan, & Winner, 2016; Snapper, Oranç, Hawley‐Dolan, Nissel, & Winner, 2015). In several experiments described in those studies, participants correctly identified the authorship of the works above chance, even after controlling for familiarity with abstract art, thus ruling out the possibility that participants consciously recognized the famous paintings. These findings suggested that there are underlying qualitative differences between professional paintings and the painted compositions made by children and animals, thereby discrediting the popular notion that abstract artworks are no better than purposeless scribbles.

Even the naïve participants in those studies reported perceiving more intention, organization, and planning in the works of trained artists (Hawley‐Dolan & Winner, 2011; Snapper et al., 2015). Out of these perceived properties, intentionality emerged as the most important predictor of authorship by a professional artist (Snapper et al., 2015). Here, we understand intentionality in the sense of goal‐directedness. Subsequently, the ability to infer intentionality refers to the capacity to judge whether an object or action has been made purposefully (Bloom, 2000; Malle, Moses, & Baldwin, 2001). Inferring intentionality is thought to be a key mechanism in assessing whether an object belongs within a certain category (e.g., a picture, a chair) and, in turn, intentionality may be embedded in, and can therefore be inferred from, the properties of artifacts such as shape, size, and texture (Bloom, 1996). In this case, the formal properties of a visual composition, such as symmetry and order, may reveal intentionality and help perceivers decide whether a picture is a work of art (Snapper et al., 2015). For that reason, it is all the more remarkable that in previous studies, people attributed high intentionality to abstract artworks, which are often thought to lack order and do not depict distinguishable elements explicitly (Gasset, 2019). One possibility is that perceived intentionality is inherent to the compositions and colors displayed in professional abstract art, but it remains to be tested if intentionality could also be found in works by non‐professional artists.

## 2 The present study

The studies discussed above (Hawley‐Dolan & Winner, 2011; Snapper et al., 2015) concluded that the participants in their experiments were able to distinguish human‐made paintings from compositions made by children and animals. However, the generalization that viewers identified a “human component” is hindered by the possibility that the perceived intentionality and organization measured in those experiments were specific features of the abstract paintings created by renowned artists. Furthermore, the participants distinguished between professional artists and paintings by children, thus hinting at differences in artworks made by humans at different developmental stages or experience with visual art. We sought to replicate these findings while focusing upon two questions: (1) Whether participants would be able to distinguish artworks by human adults untrained in the visual arts from formally similar paintings by non‐human artists and, if so, (2) what perceptual differences contributed to this distinction. We built upon the studies by Hawley‐Dolan & Winner (2011) and Snapper et al. (2015) but took a novel approach with three main differences, each of which is discussed below: stimuli sample, stimulus selection and treatment, and focus variables.

The first difference, in line with our research questions, relates to our comparative sample. Like our predecessors, we wanted to test if there was an implicit perceivable quality in human‐made abstract paintings. Therefore, we reasoned that similar works made by other intentional agents, such as great apes, should provide a good comparison. Under human encouragement, captive apes are known to sometimes engage in drawing and painting activities, and their visual output has received scholarly and media attention over the past hundred years, like in the case of the famous Congo the chimpanzee, whose works have been exhibited in high‐end galleries and sold for thousands of dollars (Morris, 2019). Therefore, the literature has developed some notions about the production and perception of art made by apes (Lenain, 1999; Martinet & Pelé, 2021; Morris, 1962; Seghers, 2014). Although a lengthy discussion of “chimpanzee art” is beyond our present scope, we should mention some relevant points related to intentionality and formal features. Regarding the former, the literature often reports that great apes appear to lack the ability to express intentionality in visual art (De Smedt & De Cruz, 2011; Preissler & Bloom, 2008; Seghers, 2014). In the artistic domain, intentionality is defined as a sense of purpose and planning, not only about what the final artwork should look like but also about its completion, that is, “knowing” when the work is finished (Seghers, 2014). Chimpanzees often lose interest in their paintings and even destroy or eat them, which might indicate their acts of drawing are less goal‐oriented and more playful than humans’ (De Waal, 2001; Martinet et al., 2021; Schiller, 1951). Nevertheless, recent studies have found that chimpanzee drawings do display a degree of intentionality and direction similar to those of young human children (Martinet et al., 2021; Martinet et al., 2023; Saito et al., 2014), with some individuals showing high levels of motivation, attention, and explorative play when engaged in drawing (Grunauer & Walguarnery, 2018; Martinet & Pelé, 2021; Martinet et al., 2023). In fact, drawing and painting in analogue and digital formats have been put forward as potential sensory enrichment activities for captive apes, providing them with focus, enjoyment, and tension release (D'Amore, Úbeda, Ballesta, & Llorente, 2015; Grunauer & Walguarnery, 2018). Likewise, it has been suggested that ape‐made art might possess incipient aesthetic sensitivity. The early ethologists interested in the biology of art already reported features of symmetry and balance in chimpanzee paintings (Morris, 1962; Schiller, 1951). These reports are echoed in current research suggesting that chimpanzee drawings are not at all random but show consistency in the use of line, color, and spatial distribution (Martinet et al., 2021, 2023). It was precisely these potential similarities and differences in intentionality and aesthetics between chimpanzee and human abstract art that motivated our interest in comparing the two samples. As for the human‐made paintings, unlike the previous studies, our stimuli did not consist of works by well‐known artists such as Karel Appel and Mark Rothko, but of paintings created by adults untrained in the visual arts, which may be more comparable to the works by great apes. It is worth clarifying that our study remained agnostic as to the objective aesthetic quality of the artworks, as well as regarding the agency, creativity, and intention of the authors, either human or ape. Although we acknowledge the relevance of these topics for a greater understanding of creativity and cognition (Sueur, Lombard, Capra, Beltzung, & Pelé, 2024), our aim for now was only to explore whether adult human observers were able to identify conspecific authorship, and if so, how.

The second difference between our experiment and the previous studies relates to stimulus selection and treatment. We controlled for and assessed the contribution of low‐level features in the performance of our participants. To begin with, we refrained from using subjective criteria to create a balanced sample of paintings. Instead, we applied an objective formal approach, selecting stimuli from a wider sample of human‐ and ape‐made paintings based on their similarity in terms of the objective measurements of low‐level features such as brightness (gray value) and entropy (more details under *Materials* in *Methods*). In addition, we standardized the low‐level features of all our stimuli. Such standardization allowed us to assess how texture and saturation contributed to participants’ performance in distinguishing human‐ and ape‐made paintings. Such differences could (unconsciously) indicate the authorship of a painting. For example, participants might notice that human paintings were oil‐based, whereas ape paintings were water‐based. Furthermore, low‐level features have been shown to affect how people perceive and judge artworks (Iigaya, Yi, Wahle, Tanwisuth, & O'Doherty, 2021; Martinet & Pelé, 2021; Mitrovic, Hegelmaier, Leder, & Pelowski, 2020; Van Geert & Wagemans, 2020; Zhang et al., 2011), and thus we considered that minimizing the differences would offer a fairer comparison of the samples.

Third, we aimed to go beyond the scope of the previous studies and made a first attempt at distilling the “human component” underlying people's ability to distinguish between human‐made art and compositions produced by non‐human artists by measuring perceptual properties that may support such distinction. As mentioned above, intentionality is pinpointed as an important factor in classifying visual art as human‐made but has not yet been properly operationalized. A review of the empirical aesthetics literature yielded balance, complexity, and organization as three key features that were recognized as visually salient, fundamental to aesthetic appreciation (Locher, Stappers, & Overbeeke, 1998; Van Geert & Wagemans, 2020), and seemingly related to perceived intentionality in relation to visual stimuli (Hawley‐Dolan & Winner, 2011). We therefore focused on these three as possible predictors of (1) intentionality ratings and (2) the classification of a painting as human‐made. Balance is the relationship between order and complexity and is defined by the weight distribution of the elements of a stimulus about its axes (Locher et al., 1998; Van Geert & Wagemans, 2020). Complexity encompasses the quantity and variety of information in a stimulus (Van Geert & Wagemans, 2020). Finally, organization (also called order or structure in the literature) refers to the structure of the information in the stimulus, such as the location of elements in the field of the areas of visual weight (Locher et al., 1998; Van Geert & Wagemans, 2020). In previous studies (Hawley‐Dolan & Winner, 2011; Snapper et al., 2015), organization was identified as a key factor in determining human authorship. Balance and complexity, for their part, have been recognized as important characteristics of abstract paintings (Wilson & Chatterjee, 2005) and appear tightly related to aesthetic preference (Clemente, Pearce, Skov, & Nadal, 2021; Locher & Nodine, 1989; Westphal‐Fitch, Huber, Gomez, & Fitch, 2012).

To address our research questions regarding authorship identification and the features that support it, we conducted two studies. Study 1 was designed to test whether participants could distinguish between abstract paintings made by non‐human great apes and artworks made by people untrained in the arts, and whether low‐level features such as color and texture aided them in distinguishing between human and non‐human authorship. Study 2 tested whether participants perceived more intentionality, balance, complexity, and organization in human artworks, as reported in the literature. In addition, Study 2 explored whether participants preferred paintings that they perceived as more intentionally made as indicated by ratings of balance, complexity, and organization. For a complete overview of the hypotheses in Studies 1 and 2, see Table 1.

**Table 1:** Summary of hypotheses tested in Studies 1 and 2

Hypotheses	Description	Study
Hypothesis 1	Participants can successfully discriminate between human‐made and ape‐made paintings above chance	1
Hypothesis 2	Participants are better at distinguishing species of authorship in the original painting condition, compared to the standardized painting condition	1
Hypothesis 3	Participants perceive human paintings as more intentionally made	2
Hypothesis 4	Participants perceive human paintings as more balanced, organized, and complex	2
Hypothesis 5	Balance, complexity, and organization are significantly associated with preference via perceived intentionality	2

## 3 Methods

### 3.1 Study 1

Study 1 aimed to test if people were able to tell apart abstract artworks made by non‐human great apes (chimpanzees) from those by human adults untrained in the visual arts. In the experiment, participants were randomly assigned to one of two conditions. The first condition consisted of 20 original paintings made by either a great ape (10 paintings) or a human (10 paintings), and participants were asked to indicate whether they thought the paintings were made by an ape or a human artist. In the second condition, the same paintings were used, only now they were standardized for saturation and texture, allowing us to control for low‐level differences. Based on previous findings, we expected participants to correctly identify the identity of the artists above chance in both conditions. Furthermore, those assigned to the original condition were expected to outperform those assigned to the standardized condition due to the additional low‐level cues present in the original paintings, such as differences in texture.

#### 3.1.1 Materials

The human‐made stimuli were extracted from a sample of abstract paintings created for a previous study (Straffon et al., 2024), in which participants were prompted to create 15 abstract paintings with a time limit of 2 min per artwork and instructed to avoid depicting any objects, people, representational motifs, or symbols. The materials were standardized and included plain A4 bond paper, a set of water‐based paints (red, yellow, blue, green, black, and white), and a medium round paintbrush (Straffon et al., 2024).

The ape‐made paintings were taken from the Schretlen animal art collection (Schretlen, 2018) housed at the Naturalis Biodiversity Center in Leiden, the Netherlands. We selected the stimuli from a total of 92 paintings (55 by humans, 37 by great apes), and 20 paintings were eventually used in the experiment (10 human‐made, 10 chimpanzee‐made). The selection was structured into multiple phases. Paintings were first equalized for size (3:2) and resolution (600 × 400 pixels). Any tags or signatures were removed using Microsoft Paint. Next, each image was measured for entropy (Cake Image Analyzer), gray‐value (ImageJ), and number of basic colors (manually). Those 10 pairs that differed the least from each other on these factors eventually constituted the experimental sample.

**Fig. 1:**
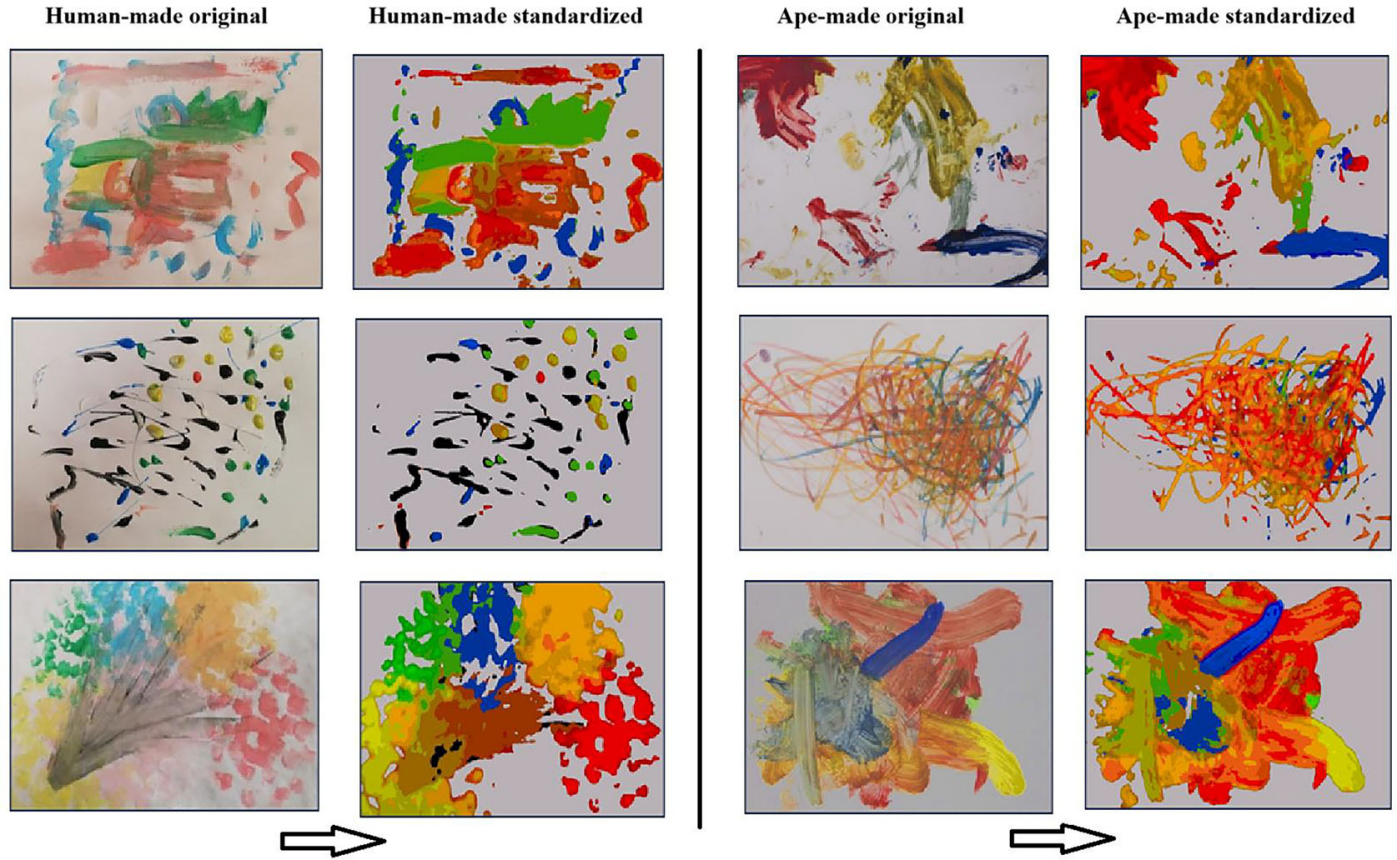
Examples of human‐made (left) and ape‐made (right) paintings used as stimuli, before and after treatment.

Although roughly equal in terms of art style, complexity, and materials, the ape‐ and human‐made paintings still differed in some low‐level aspects. For instance, human adults used water‐based paint applied with a brush, whereas great apes used oil paint applied with a brush or with hands and fingers. These differences might be used by participants to identify authorship. To control for these effects, we created an additional experimental condition in which paintings were artificially standardized for color and texture to give the impression that all the paintings were made with similar palettes and techniques. The original paintings were changed in GIMP (GIMP Development Team, 2019), using only basic colors (green, blue, yellow, red, orange, purple, and black), equal in saturation and background hue. The final palette is included in the Supporting Information (Fig. S1). Finally, all images were slightly blurred (Gaussian blur, size 1.5) to control for differences in texture (see Fig. 1). Thumbnail images of all the stimuli used in the study before and after treatment are included in the Appendix. Artworks belonging to the Schretlen collection are reproduced by permission of the Naturalis Biodiversity Center archives.

#### 3.1.2 Participants

Prior to data collection, the study was approved by the ethics committee of Leiden University. Participation was voluntary and anonymous. We recruited 72 participants via Prolific, an online platform where people can register to participate in research experiments for pay. We chose this environment over others, as Prolific has been shown to provide higher‐quality participant data (Douglas, Ewell, & Brauer, 2023). Our sample size was determined using G*power analysis set to detect large to medium‐sized effects (power set at 0.80 and error probability set at 0.05). Inclusion criteria were (1) being at least 18 years old, (2) having normal or corrected vision, and (3) not undergoing medical treatment for a mental disorder. Four participants were removed from the final dataset: one for failing the attention check, and three for duration times being extreme outliers (> 3 × interquartile range). On average, participants completed the experiment in *M* = 255,00 s. After exclusion, the sample consisted of *N* = 68 participants, with an age range from 19 to 56 years (*M* = 28.09, *SD* = 7.39), of whom 32.4% identified as male and 67.6% identified as female. The top three of nationalities were South African (31.1%), Polish (14.9%), and Portuguese (12.2%). Education level included: lower than high school (1.5%), high school graduate (25.0%), bachelor's degree (45.6%), master's degree (26.5%), and doctoral degree (1.5%). Familiarity with abstract art was ranked as 47.1% unfamiliar and 52.9% as familiar. Participants were randomly assigned to either the original condition (*n* = 34) or standardized condition (*n* = 34). There were no significant differences between conditions in terms of age (*t* = −0.326, *p* = .745), gender (*χ^2^* = 0.269, *p* = .604) or familiarity with abstract art (*χ^2^* = 0.944, *p* = .331).

#### 3.1.3 Procedure

After registering in Prolific, participants were directed to the experiment in Qualtrics. There, they received information about the purpose of the study, inclusion criteria, research design, and confidentiality, and were asked to give their informed consent. If participants did not meet the criteria or refused consent, they were directed back to Prolific. Otherwise, they received instructions that they would see a series of paintings made by either a great ape or an adult human, and they should decide which was which. They had two practice trials before starting the actual experiment. Participants saw each painting individually (500 × 333) on top of the screen with a white background. Image size was reduced to make sure participants would not have to scroll for the answer buttons, which were placed below the paintings. After completing the practice trials, participants were randomly assigned to one of two experimental conditions: the original paintings or the standardized paintings. Paintings were shown individually (in random order).

After assessing the 20th painting, participants faced an attention check consisting of a painting of a tree. On the next page, they had to report what they had just seen by choosing among five options including the correct answer and four distractors. Finally, they answered some demographic questions, as well as two questions regarding their knowledge of and familiarity with abstract art on a 5‐point scale, from “none” to “much.” On completion, participants were debriefed on the objectives of the study and were asked to give consent to the use of their data. Participants could end the experiment by going to the next page, which directed them back to Prolific.

#### 3.1.4 Analysis

First, a performance score was calculated for each participant. This process was the same for both conditions. The number of correctly identified paintings was divided by the total number of paintings, which resulted in a score that was expressed as a proportion.

**Hypothesis 1**. To test whether participants could successfully discriminate between human‐made and ape‐made paintings above chance, we conducted an exact binomial test.

**Hypothesis 2**. To test if performance scores were higher in the original condition, we tested whether standardizing the paintings hindered correct guessing of species authorship. We applied a generalized linear mixed model with species of authorship, treatment (standardized or original), and their interaction as predictors and proportion of correct responses (allowing intercepts to vary depending on stimulus and participant).

#### 3.1.5 Results

**Hypothesis 1**. A binomial test evaluated whether the observed proportion (0.69) differed significantly from the null hypothesis value of 0.50. The proportion was significantly greater than chance (*p* < .01; 95%; Confidence interval CI = 0.67−1.00). Participants were thus able to distinguish between human‐made and ape‐made paintings above chance. These findings supported Hypothesis 1.

**Fig. 2:**
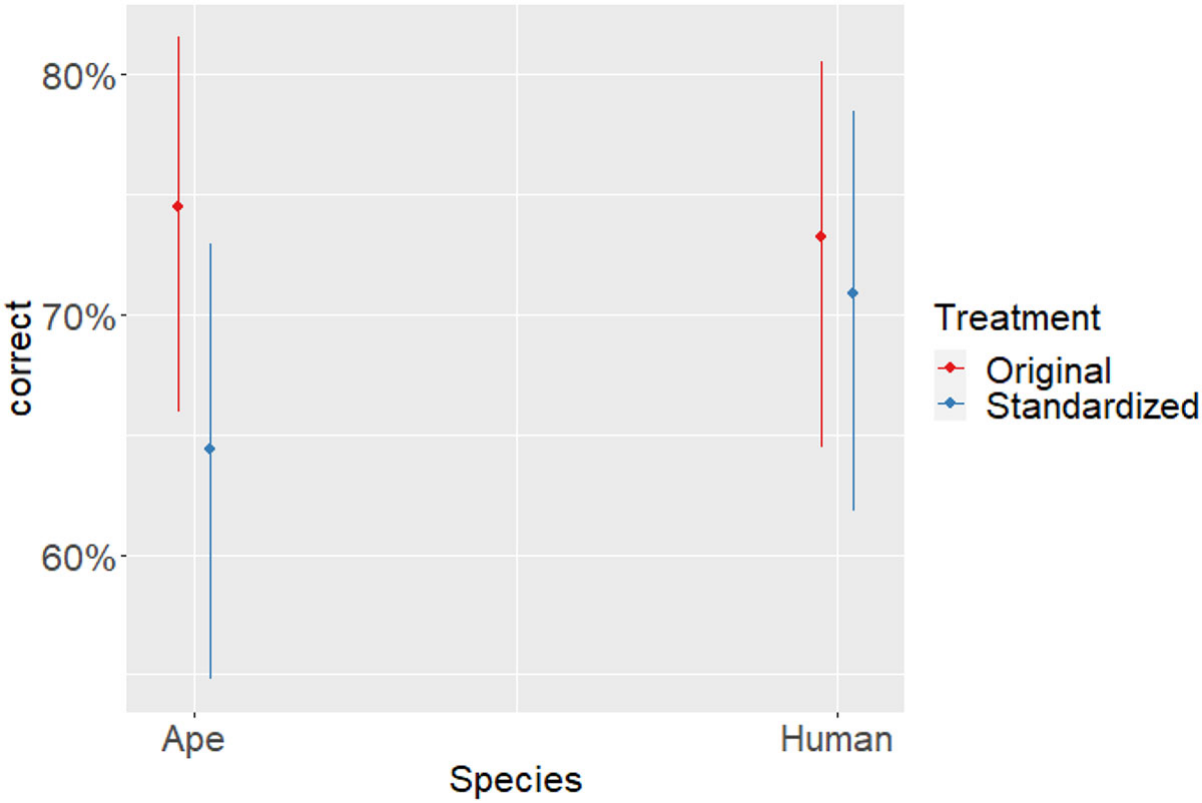
It was considerably easier for participants to correctly guess the authorship of ape‐made paintings when these were not standardized. In human‐made paintings, the difference between standardized and original paintings was much smaller. Vertical bars represent 95% CI.

**Hypothesis 2**. The model revealed that it was harder to correctly guess species of authorship in standardized stimuli (β = −0.48; *SE* = 0.21; *p* = .023; Fig. 2). However, neither species of authorship (β = −0.07; *SE* = 0.27; *p* = .807) nor the interaction between species and treatment (β = 0.36; *SE* = 0.25; *p* = .140) reached significance. Despite not reaching significance, it is interesting to point out that visual inspection of Fig. 2 suggests that the standardized condition particularly reduced classification accuracy for ape‐made paintings. Regardless, because standardization had a significant effect on classification accuracy, Hypothesis 2 was supported. These results are shown in Fig. 2, and a full model output is presented in Table 2 (output from sjPlot::tab_model).

**Table 2:** Summary of results for Hypothesis 2, testing whether standardizing color and texture hindered the ability of participants to identify whether paintings were made by humans or apes

Predictors	Odds Ratios	CI	*p*
(Intercept)	2.92	1.93–4.41	**<.001**
Human	0.94	0.55–1.58	.807
Standardized	0.62	0.41–0.94	**.023**
Human:Standardized	1.44	0.89–2.32	.140
Random Effects			
σ^2^	3.29		
τ_00 ResponseId_	0.26		
τ_00 stimulusID_	0.20		
Interclass correlation coefficient	0.12		
N _ResponseId_	68		
N _stimulusID_	20		
Observations	1360		
Marginal *R* ^2^/conditional *R* ^2^	0.009/0.130		

*Note*. This table reports odds ratios (output of function tab_model from sjPlot). Model estimates output with summary are reported in the text.

#### 3.1.6 Discussion

The results of Study 1 showed that participants were able to successfully distinguish between formally similar abstract paintings made by humans and non‐human great apes. This supports the hypothesis that people can detect the presence, or absence, of a “human component” in abstract paintings as concluded by Hawley‐Dolan & Winner (2011) and Snapper et al. (2015) based on stimuli by renowned abstract artists. Here, we extended their results by demonstrating that the effect is also found when using paintings made by humans untrained in the visual arts.

We further tested whether the ability to identify authorship was impacted when the two types of stimuli were more similar, by standardizing their color and texture. We suspected that low‐level differences between the sample sets might help participants distinguish between human‐made and ape‐made paintings. We found that the probability of correctly guessing authorship decreased slightly in the standardized condition, compared to the original condition. Thus, Hypothesis 1 held even after controlling for low‐level properties such as saturation and texture, supporting Hypothesis 2. Although species of authorship and the interaction between species and treatment were not significant, there was some difference in the average percentage of correct guessing between standardized and original ape‐made paintings when compared to the human‐made paintings. This suggests that low‐level properties were more helpful for participants in deciding whether a painting was made by an ape.

### 3.2 Study 2

Study 2 aimed at distilling the human signature that potentially enabled participants to discriminate between human‐made and non‐human‐made artworks. To do so, we investigated whether paintings made by untrained human artists were perceived as more intentionally made, since intentionality had been previously proposed as a construct that people associated with human‐made art (Snapper et al., 2015). Additionally, we wanted to test if perceived intentionality was related to perceptual properties such as balance, complexity, and organization. Last, because past studies found that human raters preferred artworks that ranked higher in intentionality (Hawley‐Dolan & Winner, 2011; Snapper et al., 2015), we also tested whether there was a relationship between perceived intentionality and preference. More specifically, we believed the low‐level features of balance, complexity, and organization mediated triggered inferences about intentionality, with intentionality driving aesthetic judgments, leading to greater ratings of preference. Thus, even though our study design precluded a fully conclusive causal interpretation, we ran a mediation analysis to explore whether our low‐level visual features related to preference both directly and indirectly (via intentionality). The hypotheses tested in Study 2 (H3, H4, H5) can be found in Table 1.

#### 3.2.1 Materials

We used the same stimuli as in Study 1, consisting of 20 paintings (10 human‐made, 10 ape‐made). As before, a standardized condition was added to assess the impact of non‐targeted low‐level differences between the works of humans and great apes.

#### 3.2.2 Participants

We recruited a new set of participants, *N* = 102 via Prolific. This sample size was in the range of previous studies (Hawley‐Dolan & Winner, 2011; Snapper et al., 2015) and was sufficient according to G*power to detect the large to medium‐sized effects (power set at 0.80 and error probability set at 0.05). Seven participants were removed from the dataset because they did not finish the study (*n* = 5), did not give consent (*n* = 1), or took an extremely long or short time to finish the experiment (*n* = 1).

On average, participants finished the experiment in *M* = 692.28 s (*SD* = 349.46). Ages ranged from 19 to 66 years (*M* = 29.50, *SD* = 8.49), 55.8% identified as male, 43.2% identified as female, and 1.0% as third gender. Nationalities that were most common were: Polish (20.4%), South African (18.4%) and Portuguese (14.3%). Education level included: lower than high school (1.1%), high school graduate (36.8%), bachelor's degree (46.3%), master's degree (13.7%), and doctoral degree (2.1%). Furthermore, 34.4% were unfamiliar and 65.6% were familiar with abstract art. There were no significant differences between conditions in terms of age *F* (3, 91) = 1.47, *p* = .227), gender (*χ^2^* = 5.95, *p* = .429) or familiarity with abstract art (*χ^2^* = 1.25, *p* = .741).

#### 3.2.3 Procedure

The registration process, access to the study, inclusion criteria, and consent information were all the same as in Study 1. After giving consent, participants were randomly assigned to one of the two experimental conditions, original or standardized. Instructions for both conditions were the same. Participants were told they would see several paintings and that they should rate them according to preference and four characteristics: intentionality, balance, complexity, and organization. Each of these was briefly explained to avoid misinterpretations. Participants first completed two practice trials before moving on to the experiment, which consisted of 20 paintings, as before. Paintings were shown individually (400 × 266). In each case, participants had to indicate in a scale (0–100) the extent to which they agreed with the following statements: (1) I like this painting, (2) I find this painting balanced, (3) I find this painting complex, (4) The artist intended to create a work of art, and (5) I find this painting organized.

Similar to Study 1, after viewing the 20th painting, participants faced an attention check, answered demographic questions, two questions regarding their knowledge of and familiarity with abstract art, were debriefed on the objectives of the study, and were asked for consent to the use of their data. By ending the experiment, participants were directed back to Prolific.

#### 3.2.4 Analysis

First, we sought to clarify how people managed to identify paintings as human‐made (Study 1). Several variables were calculated for each individual painting to be used later in a multiple regression, with “being identified as human” as the dependent (ratio) variable and various ratings (e.g., balance and complexity) as predictors.

We tested three different hypotheses (H3, H4, H5), in all cases analyzing both the original and the standardized conditions. See Table 1.

**Hypothesis 3**. To test if participants perceived human paintings as more intentionally made, we used a linear mixed effects model with Intentionality scores as response variable, and species of authorship, treatment, and their interaction as predictors, while allowing intercepts to vary depending on participant and stimulus IDs.

**Hypothesis 4**. We tested whether participants perceived human paintings as (a) more balanced, (b) more complex, and (c) more organized. We used a series of linear mixed effects models with balance, complexity, and organization scores, respectively, as the response variable, and species of authorship, treatment, and their interaction as predictors, while allowing intercepts to vary depending on participant and stimulus IDs.

**Hypothesis 5**. We tested whether the effect of compositional structure on aesthetic judgments was statistically mediated by perceived intentionality. First, we computed a principal component PC (“composition”) from balance, complexity, and organization ratings. Then, we used a bootstrapped mediation analysis (with stimulus and participant IDs as random effects) to assess whether our PC, “composition,” influenced aesthetic judgments directly and indirectly, via perceived intentionality.

#### 3.2.5 Results

**Fig. 3:**
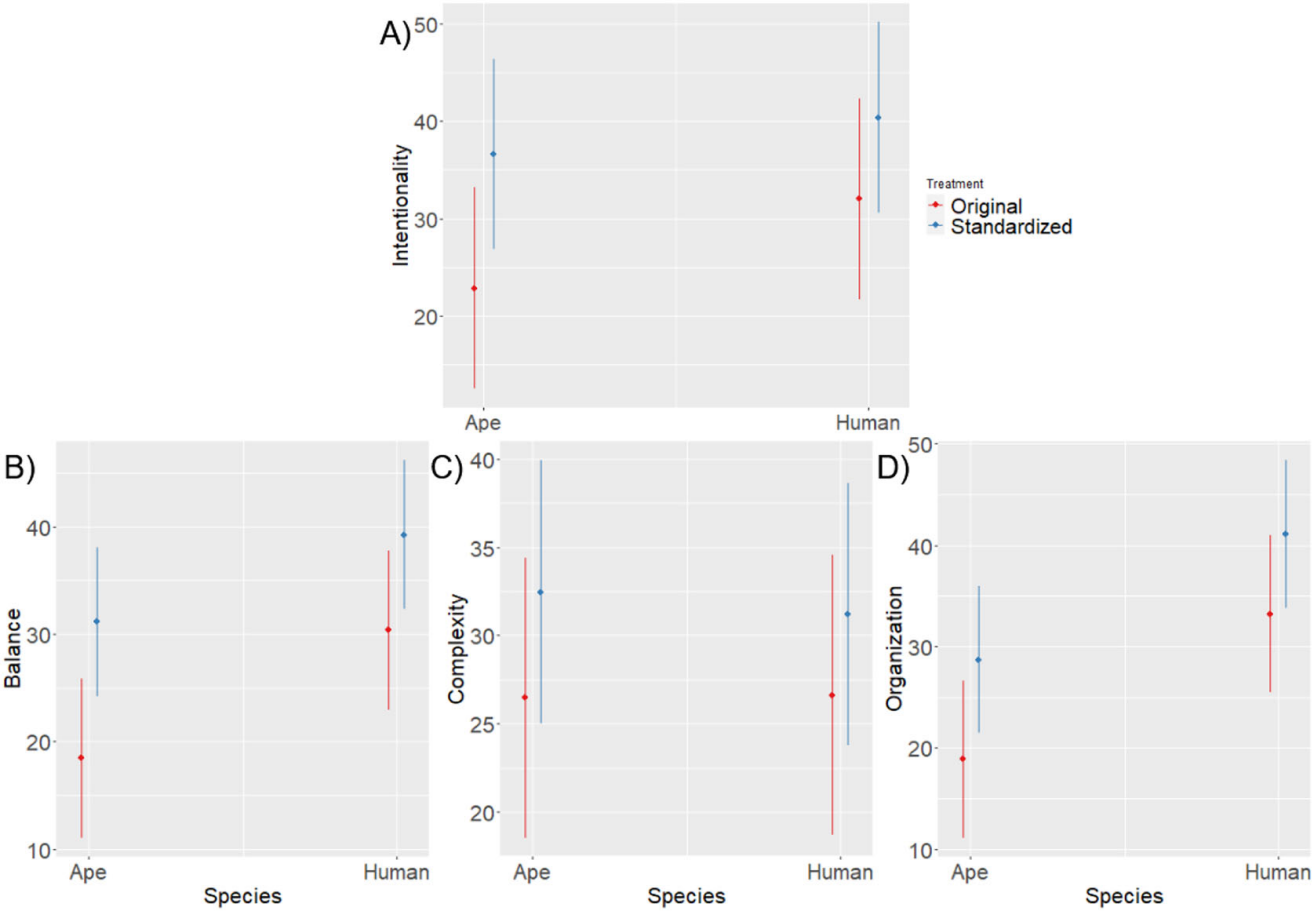
(A) Human‐made paintings were perceived as more intentionally made. Standardization resulted in more intentionally perceived paintings in both ape‐ and human‐made paintings, although the difference was greater in ape‐made paintings. (B) Human‐made paintings were perceived as more balanced. For both ape‐and human‐made paintings, the perception of balance was greater in standardized paintings. (C) Ratings of complexity were almost identical across species of authorship and between treatments. (D) Human‐made paintings were perceived as more organized. Standardization also resulted in greater perceived organization in both human‐ and ape‐made paintings. Vertical bars represent 95% CI.

**Hypothesis 3**. The results showed that human‐made paintings were perceived as significantly more intentionally made (β = 9.15; CI = 3.27–15.03; *p* = .002). Standardized paintings were also perceived as more intentionally made (β = 13.75, CI = 0.33–27.17; *p* = .045). The interaction between species of authorship and treatment was significant as well (β = −5.39; CI = −10.10–0.67; *p* = .025). See Fig. 3A and Table S1.

**Hypothesis 4**.

- Balance. We found that human‐made paintings were perceived as significantly more balanced (β = 11.90; CI = 6.74–17.05; *p* < .001). Standardized paintings were also perceived as more balanced (β = 12.69, CI = 2.59–22.79; *p* = .014). The interaction between species of authorship and treatment was not significant. See Fig. 3B and Table S2.
- Complexity. There was no difference in perceived complexity between human‐made and ape‐made paintings (β = 0.13; CI = −6.89 ‐ 7.15; *p* = .972). Treatment also had no effect on the perception of complexity (β = 5.99, CI = −4.85–16.82; *p* = .279). The interaction between species of authorship and treatment was not significant. See Fig. 3C and Table S3.
- Organization. Results showed that human‐made paintings were perceived as significantly more organized (β = 14.34; CI = 8.70–19.97; *p* = .001). Treatment neared significance as a predictor of organization (β = 9.81, CI = −0.77–20.39; *p* = .069). The interaction between species of authorship and treatment was not significant. See Fig. 3D and Table S4.

Tables S1 to S4 are found in the Supporting Information.

**Fig. 4:**
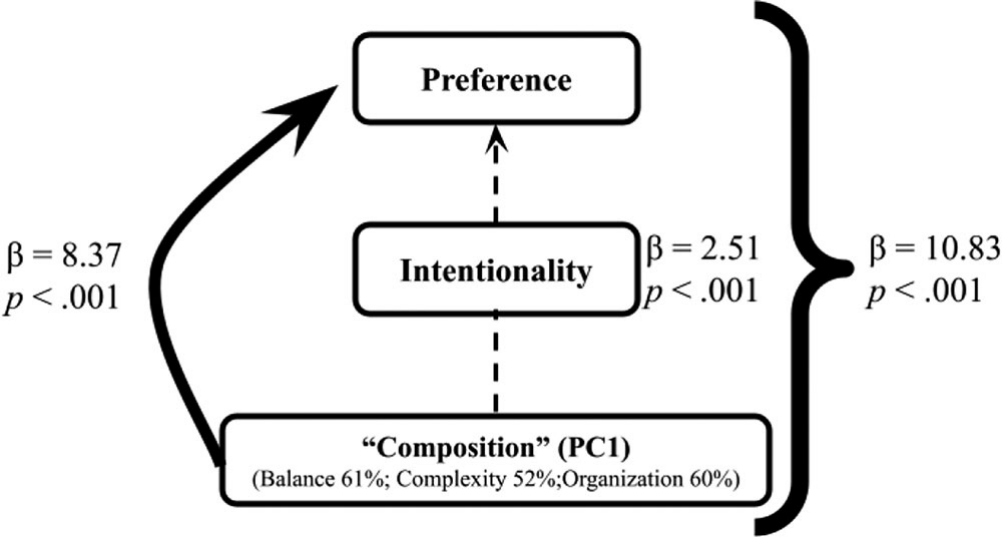
Path diagram illustrating direct (arrow with continuous line) and indirect (arrow with discontinuous line) effects on preference. The indirect effects are mediated via perceived intentionality. Total effects are displayed by the curly braces. The thickness of the lines roughly represents effect sizes.

**Hypothesis 5**. The effects of “composition” (a PC explaining 74.7% of the variance of balance, organization, and complexity) on preference were statistically mediated by perceived intentionality (β = 2.51, 95% CI = 1.41–3.55, *p* < .001). The direct effect of compositional structure was also significant (β = 8.37, 95% CI = 6.81–9.87, *p* < .001), with the total effect estimated at β = 10.83, 95% CI = 9.92–11.72, *p* < .001. These results are summarized in Fig. 4.

#### 3.2.6 Discussion

Study 2 aimed to learn more about the perceptual differences between human‐ and non‐human‐made paintings. Based on previous findings, we expected higher ratings of intentionality for the works of untrained human artists, in both the standardized and original conditions. Furthermore, we explored whether balance, complexity, and organization could significantly predict intentionality. Finally, we asked participants to rate the paintings by preference, expecting to find a positive relationship between intentionality and preference.

Overall, we found that human‐made paintings were consistently rated as higher in intentionality across both conditions, supporting Hypothesis 3, but that the perceived intentionality difference diminished when the paintings were standardized. We also found that participants saw significantly more balance and organization in human‐made paintings, compared to ape‐made paintings in the two conditions. However, there was no significant difference in perceived complexity between original human‐made paintings and ape‐made paintings in either condition. Thus, Hypothesis 4 was partially supported.

To explore the contribution of balance, complexity, and organization to ratings of intentionality and preference, we explored a principal component that we labeled “composition.” The results showed that composition was significantly associated with perceived intentionality in the paintings across conditions. Furthermore, composition was associated with preference, both directly and indirectly via intentionality, in support of Hypothesis 5. However, the direct association to preference ratings emerged as stronger.

## 4 General discussion

A series of experiments have previously demonstrated that people are able to successfully distinguish between abstract paintings made by prominent artists and similar paintings made by children or animals, on the basis of higher perceived intentionality (Hawley‐Dolan & Winner, 2011; Snapper et al., 2015). However, little has been said about *how* people infer intentionality in abstract art; therefore, it has remained unclear whether intentionality (among other properties) is exclusive to the works of renowned abstract artists, or whether it is a more general human signature. To address that gap, we aimed to replicate those experiments, but comparing abstract artworks made by human adults untrained in the visual arts to artworks made by great apes, namely chimpanzees. Moreover, we sought to explore the intrinsic and extrinsic properties that influenced people's perception of visual art as human‐made.

We carried out two online studies. In Study 1, participants observed 20 paintings and were asked to indicate whether these were made by a human or an ape. Our results supported the hypothesis that people are indeed able to identify human authorship, even when the artworks are created by individuals untrained in the visual arts. This suggests that there may be something perceptually different in human abstract art that distinguishes it from (similar) paintings made by great apes, as reported in prior research.

Study 2 aimed at finding perceptual differences that could explain the human/non‐human distinction. We asked another set of participants to rate the same sample of paintings on various attributes: intentionality, complexity, organization, balance, and preference. The results showed that people perceived human‐made paintings as more intentional, balanced, and organized but not more complex. These results suggest that balance and organization may be salient perceptual features in human‐made paintings. This is also in keeping with the literature (Hawley‐Dolan & Winner, 2011; Snapper et al., 2015).

Nevertheless, we found some notable differences between the original and standardized conditions. As a reminder, in the latter, we manipulated the color saturation and texture of the paintings to be similar across samples. Changes in these low‐level features seem to have affected participants’ answers. First, participants in the standardized condition were marginally less successful in identifying authorship than those assigned to the original condition (Study 1, H2). Second, in the standardized condition, paintings ranked higher in perceived intentionality. This difference was greater for non‐human‐made paintings (Study 2, H3). Third, paintings in the standardized condition were overall perceived as both more balanced and more organized but not as more complex (Study 2, H4). These results suggest that the manipulations of saturation and texture in the standardized condition slightly impacted participants’ decision‐making in terms of correct authorship identification (although not significantly), as well as their perception of intentionality, balance, and organization.

The effects of image treatment were partly expected, since previous research has shown the relevance of low‐level properties in overall visual art perception (Iigaya et al., 2021; Mitrovic et al., 2020; Zhang et al., 2011). However, the impact of changing saturation and texture, particularly on perceived intentionality, was somewhat unexpected. On the one hand, treatment made it harder for participants to guess authorship correctly, and on the other hand, it drove them to give higher ratings of intentionality, balance, and organization. Several factors might explain these effects. Regarding authorship recognition, some original information was lost in the process of standardization, such as stains and marks of brushes or fingers. It may be that such traces of strokes and purposeful gestures served as cues of intentionality in the original condition, making the human/non‐human distinction more difficult after treatment. Thus, by equalizing human‐ and non‐human‐made paintings in terms of saturation and texture, we might have lost certain traces of intentionality.

As for the ratings of perceived intentionality, it is possible, for example, that the elevated saturation of the standardized condition made the compositions more salient, as colors with maximal saturation are known to grab attention and be generally preferred (Camgöz, Yener, & Güvenç, 2002). The treatment also softened the strokes and the color differences, which relates to findings that people prefer smooth textures (Clemente et al., 2021) and generally favor colors with fewer associations (Van Geert & Wagemans, 2020). Since intentionality and preference are positively correlated, it is likely that saturation, texture, and intentionality may be interlinked as well.

Finally, the finding that in the standardized condition participants perceived the artworks as more balanced and organized, but not as more complex, indicates that variations in saturation and texture affected the perceived weight distribution (balance) and the structure of the elements in the stimuli (organization) but not their quantity or variety (complexity). Because organization often reduces complexity (Van Geert & Wagemans, 2020), the standardization process might have diminished complexity differences between the samples. At the same time, complex figures tend to be assessed as more meaningful (Van Geert & Wagemans, 2020), and perceived complexity seems to play a role in assessing whether a visual sign is human‐made (Salagnon, Cremona, Joliot, Errico, & Mellet, 2022). Hence, it is possible that treatment increased balance and organization resulting in reduced complexity, which may partly explain why participants found it more difficult to make the human/non‐human distinction in the standardized condition. It is also worth noting that balance and organization seem to be relevant features of visual art and graphic communication systems and may thus be seen as implicit signals of intentionality (Salagnon et al., 2022; Tylén et al., 2020; Wilson & Chatterjee, 2005). In the standardized condition, stimuli were perceived as more balanced and organized, which may in turn account for the higher rates of perceived intentionality.

Study 2 also had the aim of exploring the contribution of balance, complexity, and organization to ratings of intentionality and preference. When comprising the variance of these three key visual features as a principal component “composition,” we found a very strong direct contribution to preference and a secondary significant indirect contribution mediated by perceived intentionality. We had expected balance, complexity, and organization to influence preference due to their contribution to perceived intentionality. Although this was the case, in alignment with our Hypothesis 5, our results revealed that they more strongly contributed to preference ratings directly, which is consistent with the literature from empirical aesthetics (Locher et al., 1998; Van Geert & Wagemans, 2020).

### 4.1 Implications

In previous studies (Hawley‐Dolan & Winner, 2011; Snapper et al., 2015), intentionality emerged as a key factor used by people to distinguish between human‐made and non‐human‐made paintings, but the underlying features that helped people infer intentionality in art were not studied. Also, the relationship between intentionality and preference had been noted but remained unexplored. Here, we tested whether intentionality was inferred from three visual properties often mentioned in reference to abstract paintings: balance, complexity, and organization. In addition, we tested how these, in turn, influenced preference. We found that a composite of balance, complexity, and organization significantly predicted intentionality ratings, but complexity on its own did not differ between human and non‐human paintings, suggesting its independent role may be weaker. Thus, our research has shown explicitly for the first time that features related to the arrangement of elements in a visual composition such as distribution (balance) and structure (organization) interact to signal intentionality to observers, guiding the correct identification of an artwork as human‐made.

Our results have just touched upon what seems to be a deep and multidimensional relationship between balance, organization, and intentionality. Our species seems to be unique among primates in our ability to create and perceive regularity in visual patterns (Mühlenbeck, Liebal, Pritsch, & Jacobsen, 2016; Sablé‐Meyer et al., 2021; Westphal‐Fitch et al., 2012), making our visual system highly sensitive to variations in balance and organization (Locher et al., 1998). Furthermore, people both attribute human‐like intentionality to patterned designs and tend to perceive human‐made marks as intrinsically purposeful and meaningful (Mellet et al., 2019; Preissler & Bloom, 2008). Intentionality and organization, for instance, have been identified as universal properties of graphic systems, where increased organization amplifies saliency and perceived intentionality, making visual signs more identifiable and easier to process (Tylén et al., 2020). The high sensitivity of the human visual system toward organized stimuli may be an evolved capacity related to identifying intentionality in the context of tool and artifact‐making, since attributing human agency to crafted objects and marks may have been advantageous and necessary in early hominin visual communication (Salagnon et al., 2022).

We also started to explore the relationship between intentionality and preference. As expected, we found a positive relationship between these two constructs in both our studies and across conditions, consistent with previous reports (Hawley‐Dolan & Winner, 2011; Snapper et al., 2015). In addition, our results indicated that a composite of balance, complexity, and organization (here labeled “composition”) was strongly associated with preference for an artwork in two ways: first, directly, by contributing to visual aesthetic preferences (Westphal‐Fitch & Fitch, 2017; Wilson & Chatterjee, 2005). Second, they associate indirectly, to the extent that “composition” corresponds to increased perceived intentionality, where intentionality is also an important factor in aesthetic appreciation (Van Geert & Wagemans, 2020). This supports our proposal that people infer intentionality in non‐figurative patterns and compositions through low‐level visual properties.

In sum, our study has laid the foundation for a more systematic study on the influence of perceived intentionality in abstract art, its relationship to aesthetic preference, and the contribution that the balance (distribution), complexity (quantity and variety), and organization (structure) of elements in visual compositions make to both intentionality and preference. Despite intentionality being pinpointed in visual perception studies as a key property involved in identifying human agency and aesthetic preference in visual stimuli, it has hardly ever been operationalized. Our research constitutes an advance in this regard.

### 4.2 Limitations

The approach taken in this research intended to overcome the limitations of previous studies. For one, we used objective, replicable criteria to generate an impartial sample of human‐made and non‐human‐made paintings. In addition, we attempted to standardize the multi‐species artworks used in the experimental trials, which was of great value in creating more homogeneous visual stimuli, better suited for comparative research. Finally, we attempted to operationalize possible predictors of intentionality based on experimental data and theoretical frameworks from empirical aesthetics and comparative psychology, in order to distill the “human component” in abstract visual artworks.

Yet, our study also has some shortcomings. First, our stimuli could be improved. We acknowledge that there may be better strategies to create a balanced sample of stimuli based on objective criteria, such as using Artificial Intelligence (e.g., Beltzung, Pelé, Renoult, Shimada, & Sueur, 2022). At the time of our study, we found that the AI programs available to us were still limited in their understanding of what makes human art distinctive. But this may change in the future, in which case AI may be a useful tool for generating improved sample stimuli. Also, the chimpanzee‐made artworks in our sample were part of a larger collection of animal‐made paintings, which were created decades ago across several zoos. It is not clear whether or to what extent the human keepers intervened in the artistic activity of the animals (apart, evidently, from providing the materials). An ideal sample would involve creating human and non‐human stimuli under controlled circumstances (e.g., using similar materials) specifically for a comparative study (e.g., Martinet et al., 2021).

In addition, in Study 2, we used rating statements to measure the properties of the paintings. The use of a single statement for the different properties may have negatively affected the validity and reliability of the scores. Furthermore, it is not inconceivable that scores were affected by the anchoring bias, which occurs when responses to earlier statements influence responses to later statements (Tourangeau, 2004). And even though we explained the concepts in our statements, it may be that different individuals’ notions of intentionality, balance, complexity, and organization influenced their subjective ratings (Van Geert & Wagemans, 2020). Moving forward, it may be beneficial to apply more strict measures of perceptual features and more direct methodologies for testing subjects, such as the assessment of preference for balance (Wilson & Chatterjee, 2005), the order and complexity toolbox for aesthetics OCTA (Van Geert, Bossens, & Wagemans, 2023), or spatial index measurements applied to visual displays (Martinet et al., 2021).

Related to the previous point, our low‐level constructs of balance, complexity, and organization shared variance as revealed by high intercorrelations. To address this, we distilled the common variance into a PC that accounted for 75% across the three variables. While this approach circumvents statistical concerns pertaining to multicollinearity, it also obscures the distinct contribution of each of our original variables. Future work could examine the specific role of each of these compositional features more directly, especially in an experimental setting.

We should also mention that although our research did not directly engage either with the aesthetic quality of the artworks, or the agency, creativity, and intention of the human and chimpanzee artists, we understand these are relevant subjects in the fields of art and cognition. The debates of what constitutes art, and of what makes an artist, especially in the rise of AI, are topical issues to which studies such as the one reported here can contribute. Whereas such discussions escaped the aim of the present study, we hope our research adds to ongoing reflections on how readings of intentionality and authorship influence our definitions of human and non‐human agency, aesthetics, and artistry (Sueur et al., 2024). Finally, while conducting our studies via an online platform allowed us to recruit a large and diverse sample, we could not control the experimental environment. Ideally, future research could conduct a parallel study in the lab.

In general, more research is needed to better understand the causal relationships between different perceived properties in human visual compositions. For example, we found a positive relationship between low‐level perceptual constructs, intentionality and preference. However, it is unclear how these variables relate to each other causally. Does perceiving more intentionality lead to more preference? Or, does increased preference lead to higher perceived intentionality? Following the literature, our rationale was that intentionality constituted a higher‐level feature that might be inferred from lower‐level features, such as balance, complexity, and organization. Thus, even though our results are consistent with a mediating effect of intentionality, our data could potentially support alternative explanations. Nonetheless, following the argument by Franks, Ruxton, and Sherratt (2025) that causal inference is possible from observational data when the system is well‐understood and the assumptions are explicit, we contend that our study supports a plausible causal interpretation. While alternative explanations remain possible, the design and analytical framework (grounded in prior literature and supported by structured mediation analysis) allow us to tentatively infer that low‐level perceptual features influence aesthetic preference, in part through their effect on perceived intentionality.

## 5 Conclusion

Our study confirmed that abstract art, even when created by individuals untrained in the visual arts, transcends mere randomness. Between the strokes, the lines, and the colors that make up these paintings, people implicitly see a component of regularity and intention, which likely allows them to distinguish human‐made artworks from similar compositions made by other intentional agents, such as great apes. This suggests that, as proposed by prior research, there are some qualitative features that make up a human signature in visual compositions.

Our research has also put forward balance, complexity, and organization as three key visual features that pose a strong influence on people's preference and perception of intentionality in abstract art. This is a point that should be worth exploring in future research.

## Supporting information



Table S1. Full results of the model predicting ratings of intentionality with species of authorship, treatment, and their interaction as predictors
Table S2. Full results of the model predicting ratings of balance with species of authorship, treatment, and their interaction as predictors
Table S3. Full results of the model predicting ratings of complexity with species of authorship, treatment, and their interaction as predictors
Table S4. Full results of the model predicting ratings of organization with species of authorship, treatment, and their interaction as predictors
Figure S1. Final palette used to standardize the paintings
