# Supplementary material for: Traces of Intentionality: Balance, Complexity, and Organization in Artworks by Humans and Apes
Source: Top Cogn Sci. 2025 Sep 1;18(2):e70022. doi: 10.1111/tops.70022 (PMC13102278; doi:10.1111/tops.70022)
Supplement: Supplementary file 1 — Table S1. Full results of the model predicting ratings of intentionality with species of authorship, treatment, and their interaction as predictors Table S2. Full results of the model predicting ratings of balance with species of authorship, treatment, and their interaction as predictors Table S3. Full results of the model predicting ratings of complexity with species of authorship, treatment, and their interaction as predictors Table S4. Full results of the model predicting ratings of organization with species of authorship, treatment, and their interaction as predictors Figure S1. Final palette used to standardize the paintings [file TOPS-18-0-s001.docx]

# **Supplementary Materials**

Table S1.- Full results of the model predicting ratings of intentionality with species of authorship, treatment, and their interaction as predictors

|  | **response** | | |
| --- | --- | --- | --- |
| *Predictors* | *Estimates* | *CI* | *p* |
| *(Intercept)* | *22.84* | *12.48 – 33.19* | ***<0.001*** |
| *SpeciesHuman* | *9.15* | *3.27 – 15.03* | ***0.002*** |
| *TreatmentS* | *13.75* | *0.33 – 27.17* | ***0.045*** |
| *SpeciesHuman:TreatmentS* | *-5.39* | *-10.10 – -0.67* | ***0.025*** |
| ***Random Effects*** | | | |
| *σ^2^* | *338.09* | | |
| *τ_00_ _ResponseId_* | *513.32* | | |
| *τ_00_ _stimulusID_* | *29.57* | | |
| *ICC* | *0.62* | | |
| *N _ResponseId_* | *47* | | |
| *N _stimulusID_* | *20* | | |
| *Observations* | *940* | | |
| *Marginal R^2^ / Conditional R^2^* | *0.046 / 0.634* | | |

Table S2.- Full results of the model predicting ratings of balance with species of authorship, treatment, and their interaction as predictors

|  | **response** | | |
| --- | --- | --- | --- |
| *Predictors* | *Estimates* | *CI* | *p* |
| *(Intercept)* | *18.45* | *10.60 – 26.31* | ***<0.001*** |
| *SpeciesHuman* | *11.90* | *6.74 – 17.05* | ***<0.001*** |
| *TreatmentS* | *12.69* | *2.59 – 22.79* | ***0.014*** |
| *SpeciesHuman:TreatmentS* | *-3.84* | *-8.56 – 0.87* | *0.110* |
| ***Random Effects*** | | | |
| *σ^2^* | *337.66* | | |
| *τ_00_ _ResponseId_* | *276.38* | | |
| *τ_00_ _stimulusID_* | *19.17* | | |
| *ICC* | *0.47* | | |
| *N _ResponseId_* | *47* | | |
| *N _stimulusID_* | *20* | | |
| *Observations* | *940* | | |
| *Marginal R^2^ / Conditional R^2^* | *0.079 / 0.509* | | |

Table S3.- Full results of the model predicting ratings of complexity with species of authorship, treatment, and their interaction as predictors

|  | **response** | | |
| --- | --- | --- | --- |
| *Predictors* | *Estimates* | *CI* | *p* |
| *(Intercept)* | *26.49* | *17.43 – 35.55* | ***<0.001*** |
| *SpeciesHuman* | *0.13* | *-6.89 – 7.15* | *0.972* |
| *TreatmentS* | *5.99* | *-4.85 – 16.82* | *0.279* |
| *SpeciesHuman:TreatmentS* | *-1.41* | *-5.75 – 2.94* | *0.525* |
| ***Random Effects*** | | | |
| *σ^2^* | *286.95* | | |
| *τ_00_ _ResponseId_* | *328.06* | | |
| *τ_00_ _stimulusID_* | *50.92* | | |
| *ICC* | *0.57* | | |
| *N _ResponseId_* | *47* | | |
| *N _stimulusID_* | *20* | | |
| *Observations* | *940* | | |
| *Marginal R^2^ / Conditional R^2^* | *0.011 / 0.574* | | |

Table S4.- Full results of the model predicting ratings of organization with species of authorship, treatment, and their interaction as predictors

|  | **response** | | |
| --- | --- | --- | --- |
| *Predictors* | *Estimates* | *CI* | *p* |
| (Intercept) | 18.88 | 10.60 – 27.17 | **<0.001** |
| SpeciesHuman | 14.34 | 8.70 – 19.97 | **<0.001** |
| TreatmentS | 9.81 | -0.77 – 20.39 | 0.069 |
| SpeciesHuman:TreatmentS | -1.93 | -6.97 – 3.11 | 0.452 |
| **Random Effects** | | | |
| σ^2^ | 385.65 | | |
| τ_00_ _ResponseId_ | 301.40 | | |
| τ_00_ _stimulusID_ | 23.64 | | |
| ICC | 0.46 | | |
| N _ResponseId_ | 47 | | |
| N _stimulusID_ | 20 | | |
| Observations | 940 | | |
| Marginal R^2^ / Conditional R^2^ | 0.083 / 0.502 | | |

**Color Palette**


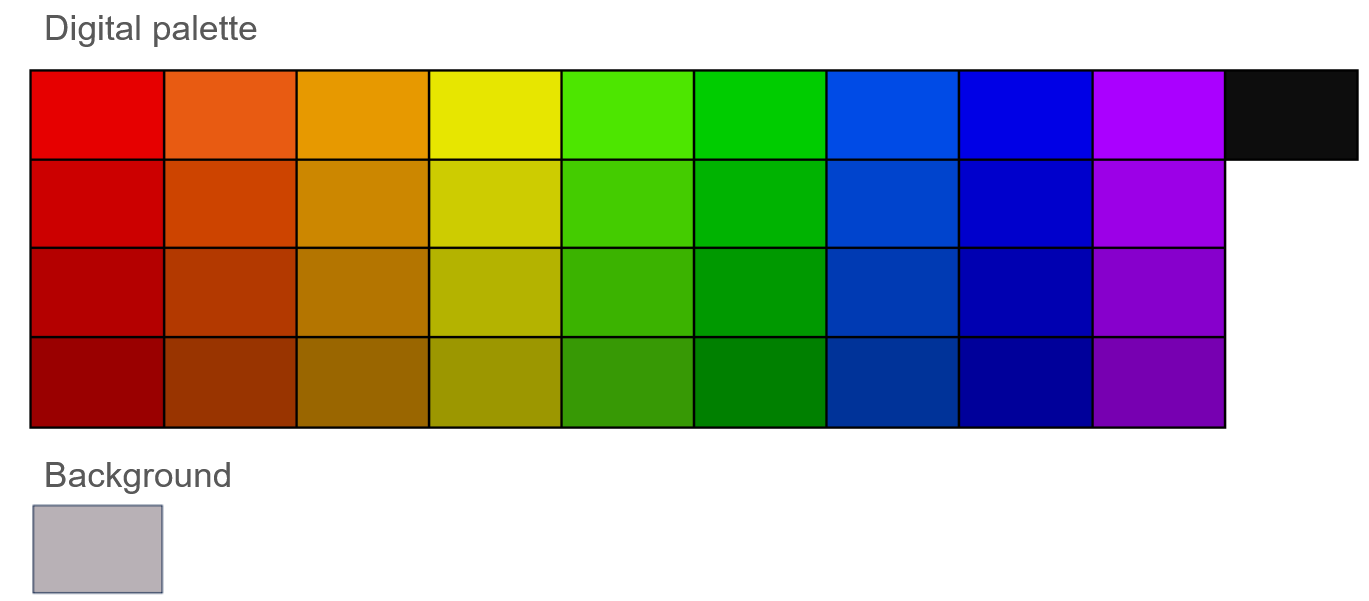


Figure S1.- Final palette used to standardize the paintings
